# A Bi-Objective Home Health Care Routing and Scheduling Model with Considering Nurse Downgrading Costs

**DOI:** 10.3390/ijerph18030900

**Published:** 2021-01-21

**Authors:** Pouria Khodabandeh, Vahid Kayvanfar, Majid Rafiee, Frank Werner

**Affiliations:** 1 Department of Industrial Engineering, Sharif University of Technology, Tehran 1365-11155, Iran; pouriakhodabandeh@ie.sharif.edu (P.K.); kayvanfar@sharif.edu (V.K.); rafiee@sharif.edu (M.R.); 2 Faculty of Mathematics, Otto-Von-Guericke-University, 39106 Magdeburg, Germany

**Keywords:** home health care, routing and scheduling, nurse downgrading, Epsilon-constraint method, bi-objective optimization

## Abstract

In recent years, the management of health systems is a main concern of governments and decision-makers. Home health care is one of the newest methods of providing services to patients in developed societies that can respond to the individual lifestyle of the modern age and the increase of life expectancy. The home health care routing and scheduling problem is a generalized version of the vehicle routing problem, which is extended to a complex problem by adding special features and constraints of health care problems. In this problem, there are multiple stakeholders, such as nurses, for which an increase in their satisfaction level is very important. In this study, a mathematical model is developed to expand traditional home health care routing and scheduling models to downgrading cost aspects by adding the objective of minimizing the difference between the actual and potential skills of the nurses. Downgrading can lead to nurse dissatisfaction. In addition, skillful nurses have higher salaries, and high-level services increase equipment costs and need more expensive training and nursing certificates. Therefore, downgrading can enforce huge hidden costs to the managers of a company. To solve the bi-objective model, an ε-constraint-based approach is suggested, and the model applicability and its ability to solve the problem in various sizes are discussed. A sensitivity analysis on the Epsilon parameter is conducted to analyze the effect of this parameter on the problem. Finally, some managerial insights are presented to help the managers in this field, and some directions for future studies are mentioned as well.

## 1. Introduction

Today, one of the main concerns of policymakers in different societies is the proper management of health systems. These systems, in addition to having a significant impact on public health, impose high costs on society. Additionally, with the decreasing birth rate and increasing elderly population, and the rise of chronic diseases, there is a growing need for health services, as countries have to spend a significant portion of their existing budget on the health area. On the other hand, due to resource constraints, planning for optimal use of the resources seems to be necessary. Furthermore, in many countries, as family members become busier and far away from each other, we see the pattern of human life moving towards an individual life, especially for the elderly, which creates the need of paying special attention to this group of people more than ever before. Therefore, one of the most effective ways to reduce the use of hospital beds and clinics is to serve patients at their place of residence. One of the most important solutions to address this problem is to establish a system for delivering efficient health care at home. Home health care (HHC) is a wide range of health care services that can be provided in the patient’s home due to illness, wound, or old age. These services are usually cheaper and more convenient than those provided in the hospital while being as efficient as the services provided in the hospital [1]. HHC services can be seen as an essential complement to health care in developed countries [2]. In this context, home service providers are often confronted with contradictions in their goals as they aim to minimize operating costs while they wish to maximize the level of offering customer service. One of the related problems that costs a lot for the companies is the optimal routing and scheduling for service providers, which has received much attention in recent years. The home health care routing and scheduling problem (HHCRSP) can be described as a dispersed collection of patients in a geographic area who require home health care, which must be provided by nurses. The HHCRSP includes designing a set of routes to deliver the scheduled care services within a planning horizon that minimizes criteria such as cost or maximizes the service quality by taking into account a number of constraints.

The problem of routing and scheduling of HHC services was first proposed by Fernandez et al. (1974) [3]. Bertels and Fahle (2006) [4], as well as Eveborn et al. (2006) [5], presented optimization methods embedded in decision support systems (DSS). Bertels and Fahle introduced a meta-heuristic approach, and Eveborn et al. introduced a heuristic method and a recurrent matching algorithm for solving the problem. Akjiratikarl et al. (2007) [6] modeled the problem as a vehicle routing problem (VRP) and used the Particle Swarm Optimization (PSO) meta-heuristic optimization method. Time window, required skills, and working time rules are common factors in most HHC routing and scheduling problems. However, the specific applications of these constraints between different papers have substantial differences from each other. In the context of when services should start, most authors have considered a hard type of time window. In addition, the soft time window can be accessed in a wide range of papers to respect the preferences of the patient, such as Bertels and Fahle (2006) [4], Eveborn et al. (2006) [5], Trautsamwieser et al. (2011) [7], Trautsamwieser and Hirsch (2011) [8], Mankowska et al. (2014) [9], Misir et al. (2015) [10], Yuan et al. (2015) [11], Braekers et al. (2016) [12]. Furthermore, several papers considered a time window for each nurse (e.g., [13,14,15,16]) which determines when a specific nurse could offer a service to patients. In addition, matching the skills of the nurses and the needs of the patients is a common feature in HHC optimization, and the domain of the skills considered may vary depending on the needs of the patients and a specific set of rules. However, in Bertels and Fahle (2006) [4], additional and non-compulsory skills are also considered as soft constraints. For example, balancing the distribution of difficult visits among all nurses is considered. In some cases, downgrading has been permitted (see, e.g., [7,8,15,17]). This means that a higher-skilled nurse can provide a lower level of service. While it provides better flexibility in the planning process and reduces travel costs, the company incurs higher costs for higher-skilled nurses. On the other hand, it can lead to a dissatisfaction of highly skilled nurses. However, in these previous studies, the huge effect of downgrading costs on an optimal planning of the HHCRSP and the role of the decision-maker was not considered. Home health care services are expensive, and company managers should decide on their acceptable downgrading level and plan their operations by considering these important downgrading cost aspects. Another issue that is considered in home health care is hourly labor law, which usually determines 5 to 10 h a day or a time window is considered. Several authors have taken a time window preference and calculated the violation by means of a penalty (see, for example, [7,8,18]). Since most papers consider the HHC routing and scheduling problem as an extension of the VRP, the main focus is on travel. However, unlike the classic VRPs, where travel distances are minimized (see, for example, Toth and Vigo (2014) [19]), in the HHC problems, the focus is often on travel costs, travel time, and the working time of the nurses. For this reason, most of the works involve overtime and waiting time (see, e.g., [7,8,10,15]). Only a few studies explicitly minimize the number of nurses at the start of the route (e.g., [11,14]). Donh et al. (2009) [13] proposed a framework based on a branch and price (B&P) algorithm for scheduling concurrent tasks. They considered HHC as a practical area that could use branch and price, but they used examples from airport operations for the computational experiments. Redjem and Marcon (2016) [20] used heuristic solution methods to manage real-size samples; they offered a two-step heuristic approach that continuously shifts jobs to meet the time constraints. Rodriguez et al. (2015) [21] considered staff dimensioning aspects of home health care in a tactical horizon to ensure that the HHC company can meet its required tasks. Spatial dimensions and a combination of nurse skills increased the complexity of their problem. In their study, demands are non-deterministic, and a two-stage integer stochastic approach is proposed. Their algorithm can give the number of nurses needed from each category without any overtime cost or external resources. Liu et al. (2016) [22] proposed a mathematical model with the consideration of lunch break requirements and decomposed it into a master problem and several pricing sub-problems. They used a branch-and-price algorithm (B&P) to solve the problem. In their solution approach, a label-correcting algorithm is applied to the lunch break constraints, and in the column generation process, some acceleration strategies are used, as well. Yuan et al. (2018) [23] proposed a daily HHCRSP considering non-deterministic travel and service times. These assumptions are derived from possible changes in the patient health status and road traffic conditions that are valid in the practical world of HHC. First, they used stochastic programming with recourse, where the recourse action is to skip patients without services if the nurse arrives later than their latest starting service time. Then, a set partitioning model is proposed, and a branch-and-price algorithm is used for solving the problem. Liu et al. (2018) [24] presented a bi-objective model to minimize the company costs and, on the other hand, to improve patient satisfaction. Decerle et al. (2019) [25] highlighted the multi-objective home health care problem with the centrality of practical planning and applied a memetic algorithm to solve it. Nasir and Dang (2018) [26] extended conventional HHCRSP to capacity and demand management aspects. To handle this problem, they proposed a mixed integer programming (MIP) model considering workload balancing, and then a heuristic method, as well as a variable neighborhood search (VNS) algorithm, were applied to solve it. Nasir et al. (2018) [27] presented a mathematical model so as to integrate resource dimensioning issues and assignment aspects considering telehealth-based care and patients’ group-based care services. Fathollahi-Fard et al. (2018) [28] presented a bi-objective green home health care model that addresses environmental pollution. Decerle et al. (2019) [25] presented an algorithm combining memetic and ant colony optimization techniques that took into account synchronization, workload balance, and time windows.

As one can see from the reviewed literature and to the best of the authors’ knowledge, most of the research did not pay any attention to downgrading cost concepts as an important home health care aspect. In some previous research, some nurses with high qualification levels were permitted to provide some usual and low-level services to patients. However, in these studies, downgrading concepts were not considered from the top-level home health care managers’ point of view and their huge downgrading costs that are enforced to their company each day. In the real-world of the home health care industry, there are various nurse skills that are very expensive, and taking their nursing certificate is complex and time-consuming. So, the companies should configure their plans to use most of the potential qualifications of their nurses. In this way, they reduce their hidden costs and increase the satisfaction level of the nurses. In this study, such requirements led us to develop conventional models to a bi-objective novel model which can engage downgrading costs into the home health care routing and scheduling problem. The managers of a home health care company can make a trade-off between the total nurse traveling times and the downgrading costs that are very costly and important for the management of home health care human resources. An Epsilon-constraint-based solution approach is presented to handle this bi-objective optimization problem. The main purpose of this approach is to provide feasible and even optimal solutions for the decision-maker. The decision-maker can adjust different values of the Epsilon parameter to analyze the effects of various downgrading levels on the objective concerning the total traveling times of the nurses and the whole planned routes.

The rest of this paper is organized as follows. Section 2 describes the problem and the mathematical model. Section 3 discusses the solution approach. Experimental results and a sensitivity analysis are presented in Section 4. Section 5 suggests some managerial insights, and finally, some conclusions and future studies are briefly presented in Section 6.

## 2. Problem Description

In general, in home health care routing and scheduling studies, researchers have always been trying to enhance the quality of services that are provided to patients while reducing the costs of the service provider. In addition to the main goals of this problem, nurse satisfaction is one of the most important and common concerns of the companies. Ignoring nurse satisfaction can cause huge hidden costs for companies.

In this problem, each of the nurses has different skill levels. The best situation for assigning nurses to patients is to use all their skills. If the company does not use some of the skills of the nurses, this could lead to nurse dissatisfaction, and this situation is called downgrading. In fact, the downgrading concept is the difference between the potential skills of the nurses and the actual skills that are used in the planning process. In addition to nurse dissatisfaction, downgrading can enforce huge hidden costs to the company. Nurses with different qualifications and skill levels have different salaries and other associated equipment costs. Thus, when some available manpower capacity of the company is not used, some huge additional costs are compelled to the company.

By investigating the previous research in this area, efforts of decreasing the difference gap between the potential skills of the nurses and their actual planned services as an optimization goal have not been observed. Therefore, in this study, the necessity of matching the potential skills of the nurses with their used skills is addressed, and a novel mathematical model is presented that aims to reduce the downgrading costs of the nurses along with reducing the total traveling time of the nurses. Home health care companies have incurred different downgrading costs by ignoring various nurse qualifications. Therefore, the value of each service type is considered as different and is determined by the company’s decision-maker. The weighted values of various nurse service types are considered by the parameter $w_{s}$. In this model, each patient requires a variety of services, where all of them must be answered by qualified nurses. Because of the sensitivity and importance of a patient’s health condition, all patients should be served in their optimal time window, and the sequence of services provided by the nurses should be respected. In addition, HHCRSP is an extended VRP problem, so it is important to consider the specific features of the VRP problem in this study. In this model, the starting and ending point of each nurse is the depot, and each nurse should depart from the patient’s place after serving him/her.

The planning of the new model presented in this study allows the decision-maker to establish a balance between a reduction of the traveling costs of the nurses in exchange for the hidden costs of not fully utilizing the skills of the nurses.

The rest of this part is organized as follows. In Section 2.1, the model assumptions are described. In Section 2.2, the model notations are introduced, and the mathematical model is presented in Section 2.3.

### 2.1. Assumptions

- Several different service needs and qualifications are included in the problem.
- All service needs of patients should be provided by qualified nurses.
- Each route of a nurse is started from the depot.
- Each nurse should end its path at the depot after visiting all planned patients.
- Each patient’s acceptable time window should be respected.
- The parameters of the patient demand, traveling time, and service time are known before the planning and considered to be deterministic.
- The correct servicing sequence of each nurse should be respected by considering the service time of the previous patient in addition to the time needed for traveling between the patients’ places.
- A single period planning strategy is considered in the problem.
- Travel sharing and multi-mode traveling concepts are not considered.
- Emergent situations and urgent service needs are not included in the problem.

### 2.2. Notations

#### 2.2.1. Subscripts


*i*
Starting node index of each travel (*i =* 1, 2, ..., *n* + 1), where *n* denotes the number of patients in the planning.
*j*
Ending node index of each travel (*j =* 2, 3, ..., *n* + 2), where *n* denotes the number of patients in the planning.
*k*
Index for the nurses (*k* = 1, 2, …, *V*), where *V* denotes the number of nurses in the planning.
*s*
Index for the services (*s* = 1, 2, …, *S*), where *S* denotes the number of different services in the planning.

#### 2.2.2. Sets


*C*
Set of patients.
*N*
Set of all nodes that includes patients and the depot.
*V*
Set of nurses.
*S*
Set of services.

#### 2.2.3. Input Parameters


*t_ij_*
Travel time between node *i* and node *j*.
*t_is_*
Required time for offering service s to patient *i*.
*l_i_*
Lower bound on the patient time window.
*u_i_*
Upper bound on the patient time window.
$a_{ks}$
Input matrix of nurse qualifications, where 1 means that nurse *k* has the qualification of doing service s. 
$g_{\mathrm{js}}$
Input matrix of patient’s service needs, where 1 means that patient *j* needs service *s*.
$w_{s}$
Weighted value of service *s* for the decision-maker.
$\varepsilon^{\prime }$
A small positive number, e.g., 0.1.

### 2.3. Decision Variables


$x_{ijks}$
1 if nurse k transfers from node *i* to *j* for offering service *s*; 0 otherwise.
$S_{iks}$
Starting time of offering service *s* to patient *i* by nurse *k*.
$b_{ks}$
1 if service *s* of nurse *k* is used in the optimal planning; 0 otherwise.

### 2.4. The Mathematical Model

#### 2.4.1. Objective Function

(1) $$f1:\;Min\;z=\;\sum_{i\in N}\sum_{j\in N}\sum_{k\in V}\sum_{s\in S}t_{ij}.x_{ijks},$$

(2) $$f2:\;Min\;z=\sum_{k\in V}\left(\sum_{s\in S}w_{s}a_{ks}-\sum_{s\in S}w_{s}b_{ks}\right).$$

The first objective function is stated in Equation (1) and minimizes the total traveling time of all routes of the nurses, and the second objective function that minimizes the downgrading costs of the nurses is presented in Equation (2).

#### 2.4.2. Constraints

(3) $$\sum_{i\in C}\sum_{s\in S}x_{1iks}=1\;\forall k\in V,$$

(4) $$\sum_{i\in C}\sum_{s\in S}x_{i1ks}=1\;\forall k\in V.$$

Constraints (3) and (4) are used to guarantee that the starting and ending place of each nurse is the depot.

(5) $$\sum_{i\in N}\sum_{s\in S}x_{ijks}-\sum_{i\in N}\sum_{s\in S}x_{jiks}=0\;\forall j\in C,k\in V.$$

Constraint (5) ensures that each nurse should depart from the patient’s place after giving care and go to another patient’s home.

(6) $$S_{iks_{1}}+t_{is_{1}}+t_{ij}-M(1-x_{ijks_{2}})\leq S_{jks_{2}}\;\forall i,j\in N,k\in V,s_{1}\in S,s_{2}\in S.$$

Constraint (6) states that a new service should be started after the time of finishing the previous service in addition to the required time for transferring the nurse to the new place.

(7) $$l_{i}\leq S_{iks}\leq u_{i}\;\forall i\in C,k\in V,s\in S.$$

Constraint (7) indicates that each patient has an acceptable time window and that the starting time of a patient’s service should be between the minimum and maximum of this time window.

(8) $$\sum_{k\in V}\sum_{i\in N}a_{ks}.x_{ijks}=g_{js}\;\forall j\in C,s\in S.$$

Constraint (8) is used to guarantee that, if patient *j* requires service *s*, exactly one of the nurses with the required qualifications should go to the patient’s place and give him/her service *s*.

(9) $$x_{ijks}=a_{ks}.g_{js}\;\forall i,j\in N,k\in V,s\in S.$$

Constraint (9) ensures that for giving service *s* by nurse *k* to patient *j*, the nurse must have the qualifications of service *s,* and the given patient must need this service.

(10) $$\varepsilon^{\prime }.\sum_{i\in N}\sum_{j\in N}x_{ijks}\leq b_{ks}\leq \sum_{i\in N}\sum_{j\in N}x_{ijks}\;\forall k\in V,s\in S.$$

Constraint (10) states that, if service *s* of nurse *k* is used at least one time in the planning, the decision variable $b_{ks}$ will be one, and otherwise, it will be zero.

(11) $$b_{ks}\leq a_{ks}\;\forall k\in V,s\in S.$$

Constraint (11) indicates that, if the nurse *k* does not have the qualification of service s, the variable $b_{ks}$ must get the value zero.

(12) $$x_{ijks},b_{ks}\in \left\{0,1\right\};S_{iks}\in {\mathrm{int}}^{+};i,j\in N;k\in V;s\in S.$$

The domain of the decision variables of the problem is defined in Condition (12).

## 3. Solution Approach

### 3.1. Background

In real-world problems, the decision-maker is always confronted with conflicting goals to make his/her decision. In the home health care routing and scheduling problem as a practical problem, the decision-maker tries to make the best possible decision by balancing the goals.

**Figure 1:**
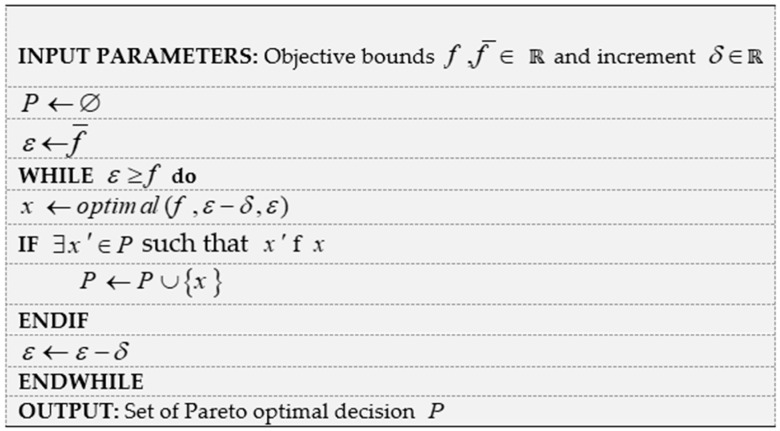
Pseudo-code of the basic Epsilon-constraint method.

The Epsilon-constraint approach is one of the most popular methods of multi-objective optimization, which attempts to optimize the most important goal by considering upper or lower limit values for the other goals. In fact, in this method, the main goal is considered as the objective function, and the other goals are added to the constraints of the model. Various elements of the Pareto front can be obtained by a systematic variation of the constraint bounds. The basic bi-objective Epsilon-constraint method is presented in Figure 1 [29].

### 3.2. Proposed Solution Approach

In this study, a bi-objective optimization method is proposed to make a trade-off between the reduction of the traveling time of the nurses and the reduction of the downgrading costs of the nurses. This method allows the decision-maker to increase the traveling time of nurses in return for reducing the downgrading costs. This solution approach is only used to obtain correct answers by considering different objectives and complex constraints of this model. Since this model is the first to consider some assumptions and HHC aspects, a comparison of these results with previous results is not possible.

In this study, the reduction of the total traveling time of the nurses is considered as the main objective function of the problem, and the second objective function that reduces the downgrading costs is contained in the constraints by considering an upper limit specified by the Epsilon parameter. In fact, the second objective function of the model is added to the constraints with an upper bound on Epsilon and is stated as Equation (13):

(13) $$\sum_{k\in V}\left(\sum_{s\in S}w_{s}a_{ks}-\sum_{s\in S}w_{s}b_{ks}\right)\leq \varepsilon .$$

The smallest amount of the Epsilon parameter can be obtained when all the skills of the nurses are used in the planning, and in this case, it is zero. On the other hand, if none of the skills of the nurses are used, this value is equal to the sum of the weighted value of the available skills, but this situation is impossible. In fact, the upper and lower limits of Epsilon would be the following values that are stated in Equation (14):

(14) $$\varepsilon_{lower}=0,\;\varepsilon_{upper}=\sum_{s\in S}w_{s}a_{ks}.$$

**Figure 2:**
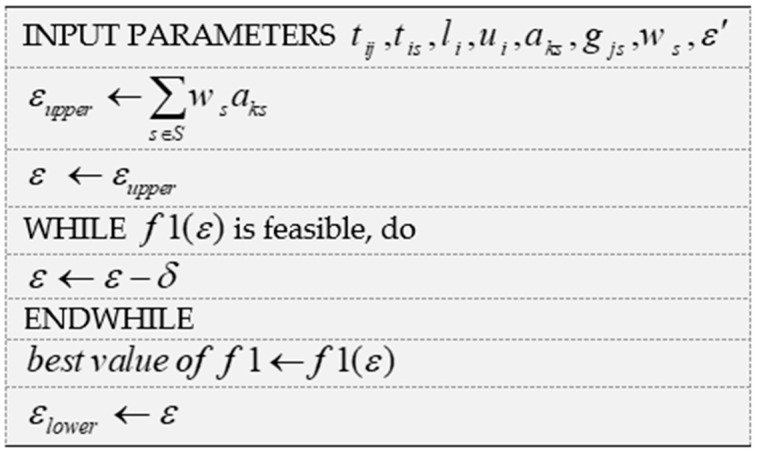
Pseudo-code of the Epsilon constraint-based approach.

To analyze the effect of changes in the Epsilon parameter on the main objective function of the problem, a heuristic approach is proposed in Figure 2 to determine the logical values of Epsilon. In this pseudo-code, the δ parameter is the reduction step of the Epsilon parameter, which is determined by the decision-maker.

## 4. Computational Experiments

In this study, in order to illustrate the effectiveness of the proposed model in the real world, the model is first tested on a small example, and the results are described, accompanied by a discussion regarding the correctness of the suggested model. Next, the model is applied to some benchmark instances in the literature taken from Mankowska et al. (2014) [9], and it is solved by IBM ILOG CPLEX Optimization Studio Version 12.6.0.0 (IBM, Armank, NY, USA). All experiments in this study are run on a computer with an Intel i7-4710HQ processor, 2.5 GHz core speed, and 8 GB of RAM.

### 4.1. Planning Process

In this subsection, a small instance is first solved to show the process of the proposed model. By doing so, the benefits of this model in contrast to traditional home health care routing and scheduling models are clarified. Table 1 shows the properties of the solved small example.

In this small example, 10 patients and 3 nurses are considered. The patients have different service needs, and the nurses have various qualifications. Each patient’s service needs and acceptable time windows are stated in Table 2.

The traveling times between the places of the patients are given in Table 3.

There are six different services and qualifications in this problem, which are presented in Table 4. Each service has a weighted value for the decision-maker. In this study, the approximate cost of each service type was first confirmed by a nursing expert after an inquiry, and then these values are scaled between 1 and 6 by the min-max scaling method to show the comparative priorities of service types. These values are rounded to discrete values for being easier to use with other problem parameters such as the Epsilon parameter.

On the other hand, each nurse has special service qualifications. The service qualifications of each nurse are explained in Table 5.

In Section 4.3, a comprehensive sensitivity analysis will be conducted on the Epsilon parameter to show the effect of the Epsilon parameter on the considered problem from different perspectives. In this section, the Epsilon value is only supposed to be equal to 10 or 7 to describe the process of planning. After solving this small example by using the proposed model, the results are shown in Table 6. In this table, the routes are illustrated by arrows and each service that is given to each patient, which is shown at the top of the patient number. In this example, the parameter Epsilon is supposed to be 10.

**Figure 3:**
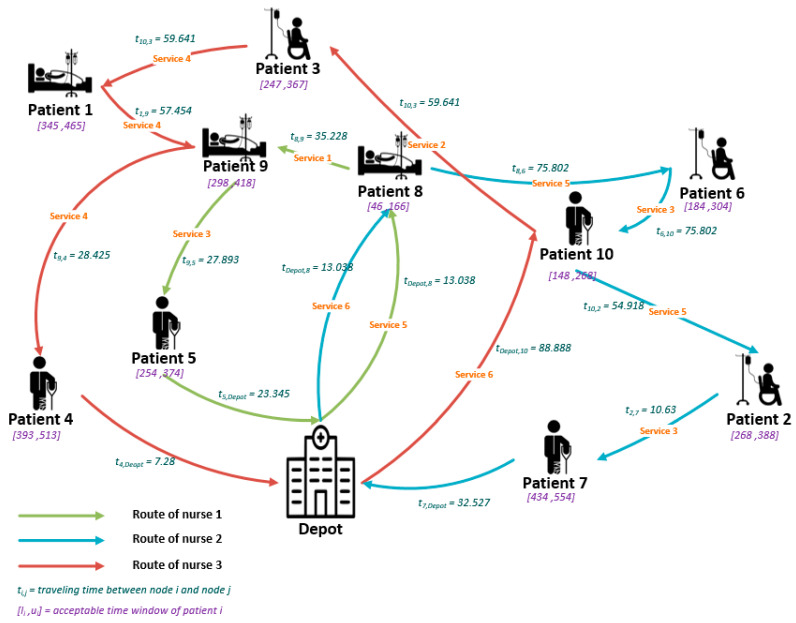
Optimal route for the small example (Epsilon = 10).

**Figure 4:**
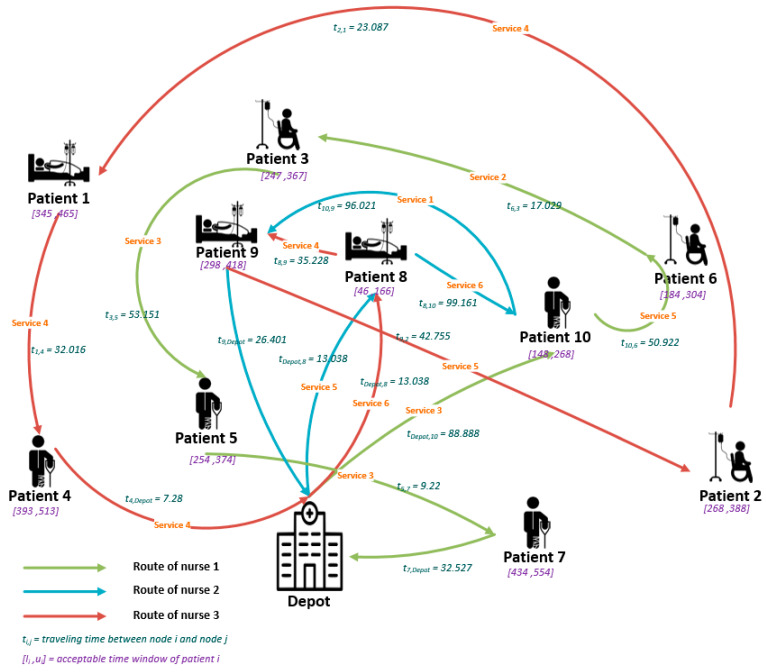
Optimal route for the small example (Epsilon = 7).

Next, a comparison between the results for two different Epsilon values is made. The results are illustrated in Figure 3 and Figure 4 for a better understanding. In Figure 3, the Epsilon value is equal to 10 and in Figure 4, the Epsilon value is supposed to be equal to 7.

As one can see from Figure 3, each patient’s service requirement is ensured, and each nurse only gives services for which he/she has the qualification. In this optimal plan, some skills of the nurses can be ignored up to the downgrading level. The ignored service qualifications are presented in Table 7, where the total weighted value of them is less than the considered Epsilon value.

In Figure 4, with decreasing the Epsilon parameter, the whole optimal routing and scheduling are affected. However, as for the previous plan, all patients’ service needs are satisfied. The ignored service qualifications of this plan are shown in Table 8, where the total weighted value of them is less than the considered Epsilon value.

By comparing the planning for the two different Epsilon values, it can be concluded that the optimal routing and scheduling can be affected severely by changing the Epsilon value.

Nurse #1 visits patients 10, 6, 3, 5, and 7 in the second planning instead of patients 8, 9, and 5 in the first one. The optimal planning for nurse #2 in the second planning proposes to serve patient 9 instead of patients 6, 2, and 7. Nurse #3 visited patients 8 and 2 in the second planning instead of patients 10 and 3 in the first one.

As it can be understood from the results (Figure 3 and Figure 4), although decreasing the Epsilon parameter can reduce the downgrading costs of the company, the summation of traveling times of the nurses will be increased from 600.43 to 639.761, and the company will have higher operational costs. So, in this context, the decision-maker should make a trade-off between reducing the downgrading costs and increasing the total traveling times of the nurses.

### 4.2. Results

In this subsection, two different categories of instances are tested to show the effectiveness of the proposed model. The first category considers 10 patients who have different service needs and acceptable time windows, 3 nurses with different qualifications, and 6 various service types. Likewise, the second category considers 25 patients who have different service needs and acceptable time windows, 5 nurses with different qualifications, and 6 various service types. The sample input parameters for each category are given in Appendix A (Table A1, Table A2, Table A3 and Table A4) and Appendix B (Table A5, Table A6, Table A7 and Table A8). The summarized properties of the benchmark instances are described in Table 9.

To the best of the authors’ knowledge, this study is the first one that considers downgrading costs in the routing and scheduling of the home health care problem. Accordingly, the value of the parameter $w_{s}$ is settled by an inquiry from a nursing expert and applying the min-max scaling method. The weighted values of different service types are presented in Table 4.

The obtained solutions for different categories of problem instances are given in Table 10 and Table 11, respectively. Further output details for the first and second instance categories are organized in Appendix C (Table A9, Table A10 and Table A11) and Appendix D (Table A12, Table A13 and Table A14).

According to the obtained results, one can conclude that the proposed bi-objective model in this paper can be well used in daily planning of home health care organizations in different sizes and can help them in their routing and scheduling decisions as well.

### 4.3. Sensitivity Analysis

#### 4.3.1. Effect of the Epsilon Parameter on the Optimal Solution Value

In this section, a sensitivity analysis of the Epsilon parameter of the model is performed to get a better insight into the effects of changing downgrading decisions on the whole model results. The chosen parameter for the sensitivity analysis is ε which determines to which amount the decision-maker is ready not to use his/her precious human resources capabilities. In fact, Epsilon is the difference between the potential skills of the nurses of the company and the actually used skills in the routing and scheduling process. The results of the sensitivity analysis of the Epsilon parameter, for instance, with 25 patients, 5 nurses, and 6 service types, are presented in Table 12.

**Figure 5:**
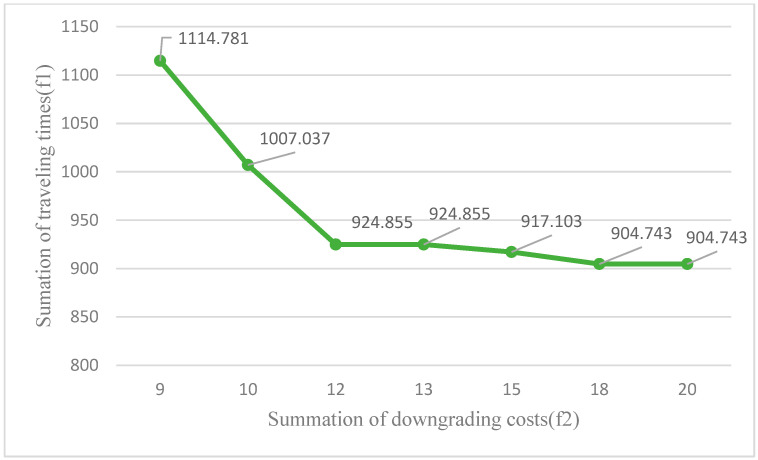
A sensitivity analysis of the results with respect to the parameter Epsilon.

The effect of changing the Epsilon parameter on the final result is illustrated in Figure 5.

From Figure 5, it can be seen that the smallest Epsilon value could be 9, and the model is infeasible for lower values. In addition, by making the Epsilon value larger than 18, the final optimal solution does not change, and 18 is an upper bound for this problem.

As it is obvious from Figure 5, the optimal solution is affected when changing the Epsilon parameter of the bi-objective model. When the downgrading costs are more important for the decision-maker, she/he reduces the Epsilon parameter to lessen the dissatisfaction of high-qualified nurses and hidden costs of the company. On the other hand, more nurse traveling time costs will be incurred to the company which is a very important aspect of home health care operational costs. In fact, the decision-maker should make a careful trade-off between reducing downgrading costs and increasing total traveling time costs.

#### 4.3.2. Upper Limit for the Meaningful Epsilon Parameter

According to the results of Section 4.3.1, it can be inferred that, if the decision-maker is willing to incur more downgrading cost, the optimal solution of the main objective function will be reduced. This section addresses the question of how much increase in the downgrading cost will continue to improve the optimal solution. An instance with 25 patients, 5 nurses, and 6 service types is used to investigate this issue. The effect of an increase of the downgrading cost on the objective function of the problem is demonstrated Table 13.

As one can see from Table 13, it can be concluded that, if the Epsilon parameter is increased to values greater than 18, there will be no effect on the total points of the difference between the potential and used qualifications of the nurses, and this violation will always remain equal to 18. This indicates that the optimal solution will not always improve as the downgrading cost increases, and there is an upper limit for the maximum amount of Epsilon, which can be considered. Therefore, if the decision-maker increases the Epsilon parameter to upper values, she/he will no longer see a change in the optimal solution because the model does not require a greater amount of mismatch between the potential and used qualifications of the nurses and has reached an optimal solution.

## 5. Managerial Insights

Some important managerial insights can be extracted from this study as follows:

1. This novel mathematical model could be used by managers for better planning of the company’s nurses considering downgrading aspects. The managers could make an appropriate trade-off between downgrading and total traveling times of the nurses. The downgrading level could be adjusted by changing the Epsilon parameter of the model.
2. In most traditional home health care routing and scheduling models, the home health care decision-maker assumes that each patient requires only one type of service. In fact, if a patient needs three services simultaneously, the plan considers it as three different patients who have the same place and health profile. The managers of the health care industry can decrease the volume of data in their companies by using this new method as well. Actually, in this plan, each patient has a unique physical and health profile, where besides removing multiple same profiles for each patient, the home health care company can have a clean and rich database from their customers. In addition, top-level managers can use this valuable resource to establish marketing strategies or manage their employees and service capacities.
3. Downgrading concepts could help the managers for making better nurse capability decisions. The managers could understand that there are some unrequired nurse qualifications in their company or there are extra needs for new skills, and he/she should hire additional skillful nurses for getting a better service level to the patients.

## 6. Conclusions and Future Studies

Health care has always been a vital concern of humans throughout history. Therefore, human societies have always tried to improve their health. Governments nowadays spend a significant portion of their budget on health. Therefore, optimization in this field has been of great interest to researchers in recent years. In general, researchers have conflicting goals in this optimization. In addition to reducing the costs, they should increase the quality of the provided services to ensure the maximum stakeholders’ satisfaction.

Due to the limited resources available in health systems nowadays, there are many concerns about providing appropriate services to patients. The capacity of hospital beds does not meet the needs of patients, and hospital admission departments are always crowded. One of the most recent ways of providing services to patients is the provision of appropriate services to the patients at their homes. Home health care can reduce unnecessary hospital admissions and make patients more comfortable. Moreover, nosocomial infections are one of the most important issues with the hospitalization of patients, which always cause many problems for the patients. These infections will be reduced by providing services to patients at home. Therefore, applying home health care in addition to reducing costs will also improve the process of providing services to patients.

One of the most important goals that have always been considered in the field of home health care is to increase the level of stakeholders’ satisfaction. Nurses are one of the most important stakeholders in this problem. Not using some of the nurses’ skills may lead to dissatisfaction of the nurses. Despite the nurses’ dissatisfaction, it would incur a hidden cost to the service provider because the plan did not use all of the potentially available resources that are very valuable to the home health care company.

In this study, a bi-objective model was proposed to minimize the downgrading costs, which characterize the difference between the potential and actual skills of the nurses, as well as to minimize the total traveling time of the nurses. In order to solve the proposed bi-objective model, an Epsilon-constraint-based solution approach was developed. In the first section of the computational experiments, the importance of the model was discussed through interpreting the obtained results obtained by solving a small example, and the applicability of the proposed model was shown. Then, the model was applied to several sets of problems, including different sizes, to confirm the efficiency of the new model for various home health care problems. Moreover, to analyze the effect of the parameter of the solution method on the problem, a sensitivity analysis was conducted on the Epsilon parameter. Finally, some managerial insights for health care managers were presented to help them to well handle their available resources.

As a direction for future research, the application of heuristic and meta-heuristic algorithms to solve larger-sized instances could be useful [30,31], specifically when exact approaches cannot be developed or are inefficient. It would be interesting to apply the proposed model to uncertain situations. Another direction could be the use of exact techniques like the branch and cut (B&C) method to solve the proposed model. Since this type of problem could be evaluated from different perspectives, owing to existing different stakeholders’ goals and proposals, adding various goals to the model could be useful. Different novel and powerful multi-objective meta-heuristic algorithms such as the multi-objective intelligent water drops (IWD) algorithm, which were proposed first by Kayvanfar et al. (2017) [32], could be applied to the model to compare the solutions obtained. Finally, developing the model using time-dependent travel times in urban regions could be another stream.

## Figures and Tables

**Table 1:** Properties of the considered small example.

Number of Patients	Number of Nurses	Number of Services
10	3	6

**Table 2:** Patients’ properties in the small example.

Patient Number	Service Needs	Acceptable Time Windows
1	S4	(345,465)
2	S5	(268,388)
3	S2	(247,367)
4	S4	(393,513)
5	S3	(254,374)
6	S5	(184,304)
7	S3	(434,554)
8	S5, S6	(46,166)
9	S1, S4	(298,418)
10	S3, S6	(148,268)

**Table 3:** Traveling times between the places of the patients in the small example.

	Depot	1	2	3	4	5	6	7	8	9	10
Depot	0	38.471	34.886	55.946	7.28	23.345	71.47	32.527	13.038	26.401	88.888
1	38.471	0	23.087	21.401	32.016	31.828	34	32.65	45.277	57.454	56.859
2	34.886	23.087	0	43.829	27.785	15.033	53.038	10.63	46.615	42.755	54.918
3	55.946	21.401	43.829	0	50.606	53.151	17.029	53.852	59.228	77.801	59.641
4	7.28	32.016	27.785	50.606	0	17.493	65.552	26.401	19.105	28.425	81.609
5	23.345	31.828	15.033	53.151	17.493	0	65	9.22	36.235	27.893	69.584
6	71.47	34	53.038	17.029	65.552	65	0	63.64	75.802	91.417	50.922
7	32.527	32.65	10.63	53.852	26.401	9.22	63.64	0	45.343	34.015	62.073
8	13.038	45.277	46.615	59.228	19.105	36.235	75.802	45.343	0	35.228	99.161
9	26.401	57.454	42.755	77.801	28.425	27.893	91.417	34.015	35.228	0	96.021
10	88.888	56.859	54.918	59.641	81.609	69.584	50.922	62.073	99.161	96.021	0

**Table 4:** Different service types in the small example.

Service ID	Service Type	Supposed Weighted Value
S1	Speech therapy	1
S2	Wound dressing	2
S3	Insulin injection	3
S4	Blood sampling	4
S5	Physiotherapy	5
S6	X-ray imaging	6

**Table 5:** Properties of the nurses in the small example.

Nurse Number	Service Qualifications
1	S1, S2, S3, S5
2	S1, S3, S5, S6
3	S2, S4, S5, S6

**Table 6:** Optimal route for the small example (Epsilon = 10).

			**S5**		**S1**		**S3**						
Nurse1	Depot	→	8	→	9	→	5	→	Depot				
			**S6**		**S5**		**S3**		**S5**		**S3**		
Nurse2	Depot	→	8	→	6	→	10	→	2	→	7	→	Depot
			**S6**		**S2**		**S4**		**S4**		**S4**		
Nurse3	Depot	→	10	→	3	→	1	→	9	→	4	→	Depot

**Table 7:** Ignored service qualifications of nurses in the small example (Epsilon = 10).

Nurse Number	Service Type Number	Supposed Weighted Value
#1	#2	2
#2	#1	1
#3	#5	5
Total weighted value		8 ≤ 10
Total traveling time		600.43

**Table 8:** Ignored service qualifications of the nurses in the small example (Epsilon = 7).

Nurse Number	Service Type Number	Supposed Weighted Value
#1	#1	1
#2	#3	3
#3	#2	2
Total weighted value		6 ≤ 7
Total traveling time		639.761

**Table 9:** Summarized properties of the benchmark instances.

Category	Instance Number	Number of Patients	Number of Nurses	Number of Services
1	#1–#10	10	3	6
2	#11–#20	25	5	6

**Table 10:** Solutions for the instances of the first category.

Instance Number	Epsilon Parameter	Optimal Solution Value
#1	10	600.43
#2	10	426.722
#3	10	602.677
#4	10	519.302
#5	10	681.19
#6	10	475.042
#7	10	357.028
#8	10	387.626
#9	10	583.52
#10	10	677.085

**Table 11:** Solutions for the instances of the second category.

Instance Number	Epsilon Parameter	Optimal Solution Value
#11	20	904.743
#12	20	823.3
#13	20	765.121
#14	20	904.989
#15	20	1833.752
#16	20	825.067
#17	20	626.793
#18	20	705.303
#19	20	1115.815
#20	20	432.561

**Table 12:** A sensitivity analysis of the results with respect to the parameter Epsilon.

Row Number	Epsilon Parameter	Optimal Solution Value
#1	9	1114.781
#2	10	1007.037
#3	12	924.855
#4	13	924.855
#5	15	917.103
#6	18	904.743
#7	20	904.743

**Table 13:** Actual used downgrading cost.

Row Number	Epsilon Parameter	Actual Used Downgrading Cost
#1	10	10
#2	12	12
#3	13	13
#4	15	15
#5	18	18
#6	20	18
#7	25	18
#8	30	18
#9	40	18
#10	50	18

## Data Availability

The data presented in this study are available on request from the corresponding author. The data are not publicly available.

## Appendix A

The properties of the sample patients for the first category instances are presented in Table A1.

**Table A1:** Sample patients’ properties for the first category instances.

Patient Number	Service Needs	Acceptable Time Windows
1	S4	(345,465)
2	S5	(268,388)
3	S2	(247,367)
4	S4	(393,513)
5	S3	(254,374)
6	S5	(184,304)
7	S3	(434,554)
8	S5, S6	(46,166)
9	S1, S4	(298,418)
10	S3, S6	(148,268)

The sample traveling times between the places of the patients for the first category instances are given in Table A2.

**Table A2:** Sample traveling times between the places of the patients for the first category instances.

	Depot	1	2	3	4	5	6	7	8	9	10
Depot	0	38.471	34.886	55.946	7.28	23.345	71.47	32.527	13.038	26.401	88.888
1	38.471	0	23.087	21.401	32.016	31.828	34	32.65	45.277	57.454	56.859
2	34.886	23.087	0	43.829	27.785	15.033	53.038	10.63	46.615	42.755	54.918
3	55.946	21.401	43.829	0	50.606	53.151	17.029	53.852	59.228	77.801	59.641
4	7.28	32.016	27.785	50.606	0	17.493	65.552	26.401	19.105	28.425	81.609
5	23.345	31.828	15.033	53.151	17.493	0	65	9.22	36.235	27.893	69.584
6	71.47	34	53.038	17.029	65.552	65	0	63.64	75.802	91.417	50.922
7	32.527	32.65	10.63	53.852	26.401	9.22	63.64	0	45.343	34.015	62.073
8	13.038	45.277	46.615	59.228	19.105	36.235	75.802	45.343	0	35.228	99.161
9	26.401	57.454	42.755	77.801	28.425	27.893	91.417	34.015	35.228	0	96.021
10	88.888	56.859	54.918	59.641	81.609	69.584	50.922	62.073	99.161	96.021	0

Sample service types for the first category instances are given in Table A3.

**Table A3:** Sample service types for the first category instances.

Service ID	Service Type	Supposed Weighted Value
S1	Speech therapy	1
S2	Wound dressing	2
S3	Insulin injection	3
S4	Blood sampling	4
S5	Physiotherapy	5
S6	X-ray imaging	6

The properties of the sample nurses for the first category instances are shown in Table A4.

**Table A4:** Nurses’ properties for the first category instances.

Nurse Number	Service Qualifications
1	S1, S2, S3, S5
2	S1, S3, S5, S6
3	S2, S4, S5, S6

## Appendix B

The properties of the sample patients for the second category instances are presented in Table A5.

**Table A5:** Sample patients’ properties for the first category instances.

Patient Number	Service Needs	Acceptable Time Windows
1	S4	(345,465)
2	S5	(268,388)
3	S2	(247,367)
4	S4	(393,513)
5	S3	(254,374)
6	S5	(184,304)
7	S3	(434,554)
8	S5	(46,166)
9	S5	(298,418)
10	S5	(148,268)
11	S2	(409,529)
12	S2	(73,193)
13	S2	(157,277)
14	S4	(63,183)
15	S1	(282,403)
16	S5	(274,394)
17	S3	(152,272)
18	S5, S6	(222,342)
19	S5, S6	(276,396)
20	S4, S6	(29,149)
21	S5, S6	(416,536)
22	S2, S4	(332,452)
23	S2, S6	(190,310)
24	S1, S4	(59,179)
25	S1, S4	(434,554)

The sample traveling times between the places of the patients for the second category instances are given in Table A6.

**Table A6:** Sample traveling times between the places of the patients for the second category instances.

	Depot	1	2	3	4	5	6	7	8	9	10	11	12	13	14	15	16	17	18	19	20	21	22	23	24	25
Depot	0	49.041	39.205	51.088	34.713	45.88	68.264	35.468	7.28	33.838	9.22	75.007	35.735	33.615	66.483	91.302	85.212	25.06	13.601	27.857	70.349	16.125	15.264	65.276	87.321	85.024
1	49.041	0	9.899	30.871	31.145	25.06	39.051	15.524	53.535	49.01	43.932	48.918	27.166	51	18.028	43.966	53.235	24.839	45.695	28.443	40	34.482	34.986	36.797	48.27	57.428
2	39.205	9.899	0	29	26	24.207	41.146	8.544	43.909	42.755	34.059	50.606	22.361	44.553	27.803	52.924	56.921	15	36.688	21.095	42.544	25.08	25.495	38.471	54.148	59.933
3	51.088	30.871	29	0	53.824	53.151	17.263	36.878	58.009	68.447	42.297	24.331	50.606	69.857	43.105	50.22	34.132	30.364	55.902	47.011	19.416	44.385	44.147	14.318	38.013	34.366
4	34.713	31.145	26	53.824	0	12.728	67.119	17.464	34.928	18.111	36.056	76.479	4	20.224	42.72	73.682	82.873	26.627	23.707	8.544	68.542	20.025	21.024	64.405	79.246	85.907
5	45.88	25.06	24.207	53.151	12.728	0	63.82	17.464	47.011	29.155	45.541	73.6	10.296	31.385	32.14	64.008	78.294	31.575	36.056	18.028	64.9	30.083	31.048	61.4	72.732	82.377
6	68.264	39.051	41.146	17.263	67.119	63.82	0	49.659	75.107	83.169	59.54	9.899	63.506	84.77	44.721	39.85	17	46.39	72.339	61.351	2.236	60.531	60.407	3	22.023	18.788
7	35.468	15.524	8.544	36.878	17.464	17.464	49.659	0	39.051	34.366	32.202	59.059	13.892	36.222	31.401	59.481	65.46	15.033	30.414	13.038	51.078	19.849	20.518	46.957	62.394	68.447
8	7.28	53.535	43.909	58.009	34.928	47.011	75.107	39.051	0	30.265	16.492	82.055	36.715	29.614	70.434	96.607	92.087	30.676	11.402	29.411	77.162	19.209	18.601	72.111	93.744	92.087
9	33.838	49.01	42.755	68.447	18.111	29.155	83.169	34.366	30.265	0	39.446	92.114	22.091	2.236	60.638	91.788	99.459	38.588	20.248	21.84	84.77	26.173	26.87	80.324	96.747	101.843
10	9.22	43.932	34.059	42.297	36.056	45.541	59.54	32.202	16.492	39.446	0	66.008	36.056	39.661	61.847	84.599	76.42	19.105	19.849	28.018	61.66	16.031	15.033	56.569	79.12	76
11	75.007	48.918	50.606	24.331	76.479	73.6	9.899	59.059	82.055	92.114	66.008	0	72.945	93.638	54.203	45.277	12.207	54.571	80.231	70.385	9.434	68.659	68.447	12.207	23.259	10.05
12	35.735	27.166	22.361	50.606	4	10.296	63.506	13.892	36.715	22.091	36.056	72.945	0	24.187	38.897	69.721	79.12	24.597	25.807	8.062	64.885	20.224	21.213	60.828	75.313	82.292
13	33.615	51	44.553	69.857	20.224	31.385	84.77	36.222	29.614	2.236	39.661	93.638	24.187	0	62.817	93.904	101.139	39.825	20.125	23.537	86.4	27.019	27.659	81.908	98.615	103.407
14	66.483	18.028	27.803	43.105	42.72	32.14	44.721	31.401	70.434	60.638	61.847	54.203	38.897	62.817	0	32.062	54.489	42.802	61.587	42.942	44.777	51.225	51.856	43.463	45.222	60.605
15	91.302	43.966	52.924	50.22	73.682	64.008	39.85	59.481	96.607	91.788	84.599	45.277	69.721	93.904	32.062	0	38.328	66.242	89.538	72.277	38.328	78	78.39	40.853	24.515	46.271
16	85.212	53.235	56.921	34.132	82.873	78.294	17	65.46	92.087	99.459	76.42	12.207	79.12	101.139	54.489	38.328	0	63.285	89.275	77.621	15.033	77.414	77.318	20	14	8
17	25.06	24.839	15	30.364	26.627	31.575	46.39	15.033	30.676	38.588	19.105	54.571	24.597	39.825	42.802	66.242	63.285	0	26.019	18.439	48.26	14.142	14.036	43.417	63.506	64.537
18	13.601	45.695	36.688	55.902	23.707	36.056	72.339	30.414	11.402	20.248	19.849	80.231	25.807	20.125	61.587	89.538	89.275	26.019	0	19.105	74.243	12.042	12	69.354	89.275	90.255
19	27.857	28.443	21.095	47.011	8.544	18.028	61.351	13.038	29.411	21.84	28.018	70.385	8.062	23.537	42.942	72.277	77.621	18.439	19.105	0	62.936	12.166	13.153	58.524	75.24	80.056
20	70.349	40	42.544	19.416	68.542	64.9	2.236	51.078	77.162	84.77	61.66	9.434	64.885	86.4	44.777	38.328	15.033	48.26	74.243	62.936	0	62.394	62.29	5.099	19.849	17.493
21	16.125	34.482	25.08	44.385	20.025	30.083	60.531	19.849	19.209	26.173	16.031	68.659	20.224	27.019	51.225	78	77.414	14.142	12.042	12.166	62.394	0	1	57.559	77.233	78.645
22	15.264	34.986	25.495	44.147	21.024	31.048	60.407	20.518	18.601	26.87	15.033	68.447	21.213	27.659	51.856	78.39	77.318	14.036	12	13.153	62.29	1	0	57.428	77.318	78.447
23	65.276	36.797	38.471	14.318	64.405	61.4	3	46.957	72.111	80.324	56.569	12.207	60.828	81.908	43.463	40.853	20	43.417	69.354	58.524	5.099	57.559	57.428	0	24.413	21.541
24	87.321	48.27	54.148	38.013	79.246	72.732	22.023	62.394	93.744	96.747	79.12	23.259	75.313	98.615	45.222	24.515	14	63.506	89.275	75.24	19.849	77.233	77.318	24.413	0	22
25	85.024	57.428	59.933	34.366	85.907	82.377	18.788	68.447	92.087	101.843	76	10.05	82.292	103.407	60.605	46.271	8	64.537	90.255	80.056	17.493	78.645	78.447	21.541	22	0

The sample service types for the second category instances are given in Table A7.

**Table A7:** Sample service types for the second category instances.

Service ID	Service Type	Supposed Weighted Value
S1	Speech therapy	1
S2	Wound dressing	2
S3	Insulin injection	3
S4	Blood sampling	4
S5	Physiotherapy	5
S6	X-ray imaging	6

The properties of the sample nurses for the second category instances are shown in Table A8.

**Table A8:** Nurses’ properties for the second category instances.

Nurse Number	Service Qualifications
1	S1, S3, S4, S5
2	S2, S3, S6
3	S1, S2, S5, S6
4	S2, S4, S5, S6
5	S1, S3, S4, S5, S6

## Appendix C

The sample results of the decision variables $x_{ijks}$ for the first category instances are shown in Table A9.

**Table A9:** Sample results of the decision variable $x_{ijks}$ for the first category instances.

*i*	*j*	*k*	*s*	$x_{ijks}$
11	4	3	2	1
11	3	2	5	1
10	6	1	3	1
10	5	3	4	1
9	10	1	1	1
9	7	2	5	1
8	1	2	1	1
7	11	2	3	1
6	1	1	2	1
5	1	3	5	1
4	2	3	4	1
3	8	2	3	1
2	10	3	4	1
1	11	3	6	1
1	9	2	6	1
1	9	1	5	1

The sample results of the decision variables $S_{iks}$ for the first category instances are presented Table A10.

**Table A10:** Sample results of the decision variable $S_{iks}$ for the first category instances.

*i*	*k*	*s*	$S_{iks}$
9	1	5	46
10	1	1	298
6	1	3	340
9	2	6	46
7	2	5	184
11	2	3	268
3	2	5	337
8	2	3	434
11	3	6	235
4	3	2	309
2	3	4	345
10	3	4	417
5	3	4	460

The sample results of the decision variables $b_{ks}$ for the first category instances are presented in Table A11.

**Table A11:** Sample results of the decision variables $b_{ks}$ for the first category instances.

*k*	*s*	$b_{ks}$
1	1	1
1	3	1
1	5	1
2	3	1
2	5	1
2	6	1
3	2	1
3	4	1
3	6	1

## Appendix D

The sample results of the decision variables $x_{ijks}$ for the second category instances are shown Table A12.

**Table A12:** Sample results of the decision variables $x_{ijks}$ for the second category instances.

*i*	*j*	*k*	*s*	$x_{ijks}$
26	1	5	3	1
26	1	3	5	1
25	21	3	6	1
25	7	5	5	1
24	16	5	1	1
24	4	3	2	1
23	1	4	6	1
23	1	2	3	1
22	23	4	4	1
22	23	2	2	1
21	25	5	4	1
21	24	3	2	1
20	19	2	6	1
20	6	1	3	1
19	22	2	6	1
19	14	4	2	1
18	20	1	5	1
17	26	5	4	1
16	17	5	5	1
15	21	5	4	1
14	10	4	5	1
13	20	2	6	1
12	26	3	1	1
11	18	1	3	1
10	5	4	4	1
9	19	4	5	1
8	1	1	1	1
7	24	5	6	1
6	2	1	4	1
5	22	4	5	1
4	12	3	2	1
3	8	1	3	1
2	3	1	5	1
1	25	3	1	1
1	15	5	4	1
1	13	2	2	1
1	11	1	5	1
1	9	4	5	1

The sample results of the decision variables $S_{iks}$ for the second category instances are given in Table A13.

**Table A13:** Sample results of the decision variables $S_{iks}$ for the second category instances.

*i*	*k*	*s*	$S_{iks}$
11	1	5	148
18	1	3	182
20	1	5	276
6	1	3	309
2	1	4	349
3	1	5	373
8	1	3	554
13	2	2	73
20	2	6	276
19	2	6	310
22	2	6	416
23	2	2	431
25	3	1	88
21	3	6	122
24	3	2	190
4	3	2	247
12	3	2	409
26	3	1	434
9	4	5	46
19	4	5	222
14	4	2	277
10	4	5	298
5	4	4	393
22	4	5	428
23	4	4	443
15	5	4	67
21	5	4	126
25	5	4	160
7	5	5	197
24	5	6	214
16	5	1	341
17	5	5	394
26	5	4	434

The sample results of the decision variables $b_{ks}$ for the second category instances are presented in Table A14.

**Table A14:** Sample results of the decision variables $b_{ks}$ for the second category instances.

*k*	*s*	$b_{ks}$
5	6	1
5	5	1
5	4	1
5	1	1
4	5	1
4	4	1
4	2	1
3	6	1
3	2	1
3	1	1
2	6	1
2	2	1
1	5	1
1	4	1
1	3	1

## References

[1] Alodhayani A.A. (2017). Comparison between home health care and hospital services in elder population: Cost-effectiveness. Biomed. Res..

[2] Tyan M. (2010). Understanding Taiwanese Home Healthcare Nurse Managers’ Empowerment and International Learning Experiences: Community-Based Participatory Research Approach Using a US Home Healthcare Learning Tour.

[3] Fernández A., Gregory G., Hindle A., Lee A.C. (1974). A Model for Community Nursing in a Rural County. J. Oper. Res. Soc..

[4] Bertels S., Fahle T. (2006). A hybrid setup for a hybrid scenario: Combining heuristics for the home health care problem. Comput. Oper. Res..

[5] Eveborn P., Flisberg P., Rönnqvist M. (2006). Laps Care—An operational system for staff planning of home care. Eur. J. Oper. Res..

[6] Akjiratikarl C., Yenradee P., Drake P.R. (2007). PSO-based algorithm for home care worker scheduling in the UK. Comput. Ind. Eng..

[7] Trautsamwieser A., Gronalt M., Hirsch P. (2011). Securing home health care in times of natural disasters. OR Spectr..

[8] Trautsamwieser A., Hirsch P. (2011). Optimization of daily scheduling for home health care services. J. Appl. Oper. Res..

[9] Mankowska D.S., Meisel F., Bierwirth C. (2013). The home health care routing and scheduling problem with interdependent services. Health Care Manag. Sci..

[10] Mısır M., Smet P., Berghe G.V., Misir M. (2015). An analysis of generalised heuristics for vehicle routing and personnel rostering problems. J. Oper. Res. Soc..

[11] Yuan B., Liu R., Jiang Z. (2015). A branch-and-price algorithm for the home health care scheduling and routing problem with stochastic service times and skill requirements. Int. J. Prod. Res..

[12] Braekers K., Hartl R.F., Parragh S.N., Tricoire F. (2016). A bi-objective home care scheduling problem: Analyzing the trade-off between costs and client inconvenience. Eur. J. Oper. Res..

[13] Dohn A., Kolind E., Clausen J. (2009). The manpower allocation problem with time windows and job-teaming constraints: A branch-and-price approach. Comput. Oper. Res..

[14] Allaoua H., Borne S., Létocart L., Calvo R.W. (2013). A matheuristic approach for solving a home health care problem. Electron. Notes Discret. Math..

[15] Hiermann G., Prandtstetter M., Rendl A., Puchinger J., Raidl G.R. (2015). Metaheuristics for solving a multimodal home-healthcare scheduling problem. Cent. Eur. J. Oper. Res..

[16] Mutingi M., Mbohwa C. (2014). Multi-objective homecare worker scheduling: A fuzzy simulated evolution algorithm approach. IIE Trans. Health Syst. Eng..

[17] Rest K.-D., Hirsch P. (2015). Daily scheduling of home health care services using time-dependent public transport. Flex. Serv. Manuf. J..

[18] Fikar C., Hirsch P. (2015). A matheuristic for routing real-world home service transport systems facilitating walking. J. Clean. Prod..

[19] Toth P., Vigo D. (2014). Vehicle Routing: Problems, Methods, and Applications.

[20] Redjem R., Marcon E. (2016). Operations management in the home care services: A heuristic for the caregivers’ routing problem. Flex. Serv. Manuf. J..

[21] Rodriguez C., Garaix T., Xie X., Augusto V. (2015). Staff dimensioning in homecare services with uncertain demands. Int. J. Prod. Res..

[22] Liu R., Yuan B., Jiang Z. (2016). Mathematical model and exact algorithm for the home care worker scheduling and routing problem with lunch break requirements. Int. J. Prod. Res..

[23] Yuan B., Liu R., Jiang Z. (2018). Daily scheduling of caregivers with stochastic times. Int. J. Prod. Res..

[24] Liu M., Yang D., Su Q., Xu L. (2018). Bi-objective approaches for home healthcare medical team planning and scheduling problem. Comput. Appl. Math..

[25] Decerle J., Grunder O., El Hassani A.H., Barakat O. (2019). A hybrid memetic-ant colony optimization algorithm for the home health care problem with time window, synchronization and working time balancing. Swarm Evol. Comput..

[26] Nasir J.A., Dang C. (2018). Solving a More Flexible Home Health Care Scheduling and Routing Problem with Joint Patient and Nursing Staff Selection. Sustainability.

[27] Nasir J.A., Hussain S., Dang C. (2018). An Integrated Planning Approach towards Home Health Care, Telehealth and Patients Group Based Care. J. Netw. Comput. Appl..

[28] Fathollahi-Fard A.M., Hajiaghaei-Keshteli M., Tavakkoli-Moghaddam R. (2018). A bi-objective green home health care routing problem. J. Clean. Prod..

[29] Laumanns M., Thiele L., Zitzler E. (2006). An efficient, adaptive parameter variation scheme for metaheuristics based on the epsilon-constraint method. Eur. J. Oper. Res..

[30] Kolahan F., Kayvanfar V. (2009). A heuristic algorithm approach for scheduling of multi-criteria unrelated parallel machines. World Acad. Sci. Eng. Technol..

[31] Kayvanfar V., Alizadeh K.M., Teymourian E. Intelligent water drops algorithm on parallel machines scheduling. Proceedings of the 2015 International Conference on Industrial Engineering and Operations Management (IEOM).

[32] Kayvanfar V., Husseini S.M., Karimi B., Sajadieh M.S. (2017). Bi-objective Intelligent Water Drops Algorithm to a Practical Multi-Echelon Supply Chain Optimization Problem. J. Manuf. Syst..

